# High extracellular phosphate increases platelet polyphosphate content

**DOI:** 10.1080/09537104.2020.1817358

**Published:** 2020-09-06

**Authors:** Nima Abbasian, Matthew T. Harper

**Affiliations:** Department of Pharmacology, University of Cambridge, Cambridge, UK

**Keywords:** Coagulation, hyperphosphatemia, inorganic phosphate, platelets, polyphosphate, thrombosis

## Abstract

Platelet-derived extracellular polyphosphate (PolyP) is a major mediator of thrombosis. PolyP is a linear chain of inorganic phosphate (P*_i_*) and is stored in platelet dense granules. P*_i_* enters cells from the extracellular fluid through phosphate transporters and may be stored as PolyP. Here we show that high extracellular P*_i_* concentration *in vitro* increases platelet PolyP content, in a manner dependent on phosphate transporters, IP6K and V-type ATPases. The increased PolyP also enhanced PolyP-dependent coagulation in platelet-rich plasma. These data suggest a mechanistic link between hyperphosphatemia, PolyP and enhanced coagulation, which may be important in pathologies such as chronic kidney disease.

## Introduction

Platelet-derived extracellular polyphosphate (PolyP) is a major mediator of hemostasis, thrombosis, and vascular inflammation and is a promising therapeutic target [1–3]. Platelets store PolyP in their dense granules and release it during platelet activation[4]. Extracellular PolyPs enhance coagulation while also limiting fibrinolysis[1]. Platelet PolyPs therefore link platelet granule secretion to enhanced coagulation.

PolyPs are polyanionic, linear chains of inorganic phosphate (P*_i_*) found in both prokaryotic and eukaryotic cells. In bacteria, intracellular PolyP is involved in energy storage, protection from heavy metal toxicity, and as a molecular chaperone[5], although its role in eukaryotes is less well understood. Intracellular PolyP may act as buffer to minimize fluctuations in cytosolic P*_i_* concentration ([P*_i_*]_cyt_). Since P*_i_* is essential to signal transduction and energy metabolism, its levels in the cytosol must be tightly controlled. For example, endothelial cells undergo plasma membrane blebbing and release microparticles when challenged with high [P*_i_*]_ex_[6], and can undergo apoptosis[7].

Normal plasma [P*_i_*] fluctuates between approximately 1.0–1.45 mM (3.0–4.5 mg/dL phosphorus) in adults[8]. High plasma [P*_i_*] (hyperphosphatemia) can be caused acutely by excessive phosphate intake or chronically by insufficient renal excretion and contributes to the high cardiovascular risk of chronic kidney disease (CKD), which includes vascular calcification and abnormal coagulation[9]. Hyperphosphatemia may also increase [P*_i_*]_cyt_, since P*_i_* enters cells from the extracellular fluid through phosphate transporters[6]. Storing P*_i_* monomers in PolyP is one potential mechanism to reduce toxicity during high P*_i_* load. In this study, we therefore investigated the effect of high extracellular inorganic phosphate concentration ([P*_i_*]_ex_) on platelets, and specifically whether this affects platelet PolyP levels.

## Methods

Washed platelets were isolated from blood drawn from healthy drug-free volunteers with approval from the Human Biology Research Ethics Committee, University of Cambridge, as previously described[10], except that the isolated platelets were initially resuspended in HEPES-buffered saline (HBS) without P*_i_*. NaH_2_PO_4_ was then added to the following final concentrations (in mM): 0.34, which is the concentration we have previously used in our HBS; 1.0, which represents physiological serum [P*_i_*]; and 2.5, which occurs in hyperphosphatemia[8]. PolyP was separated from platelet lysates using silica spin columns[11]. DNA or RNA was removed by treating samples with DNAase and RNAase. PolyP in the samples was stained with DAPI to measure PolyP levels[12]. This fluorescence was significantly reduced by treatment with alkaline phosphatase, which degrades PolyP, by 66.9 ± 1.9% (n = 4). Clot turbidometry of PRP in response to P*_i_*-loading in the presence or absence of TRAP-6 was measured as previously described[13]. [P*_i_*]_ex_ in PRP showed variation between samples, though in none was [P*_i_*]_ex_ greater than 1.1 mM. Where [P*_i_*]_ex_ was lower than 1 mM, it was increased to 1 mM by addition of NaH_2_PO_4_. Hyperphosphatemia was mimicked by addition of NaH_2_PO_4_ to give [P*_i_*]_ex_ of 2.5 mM.

## Results

**Figure 1.**
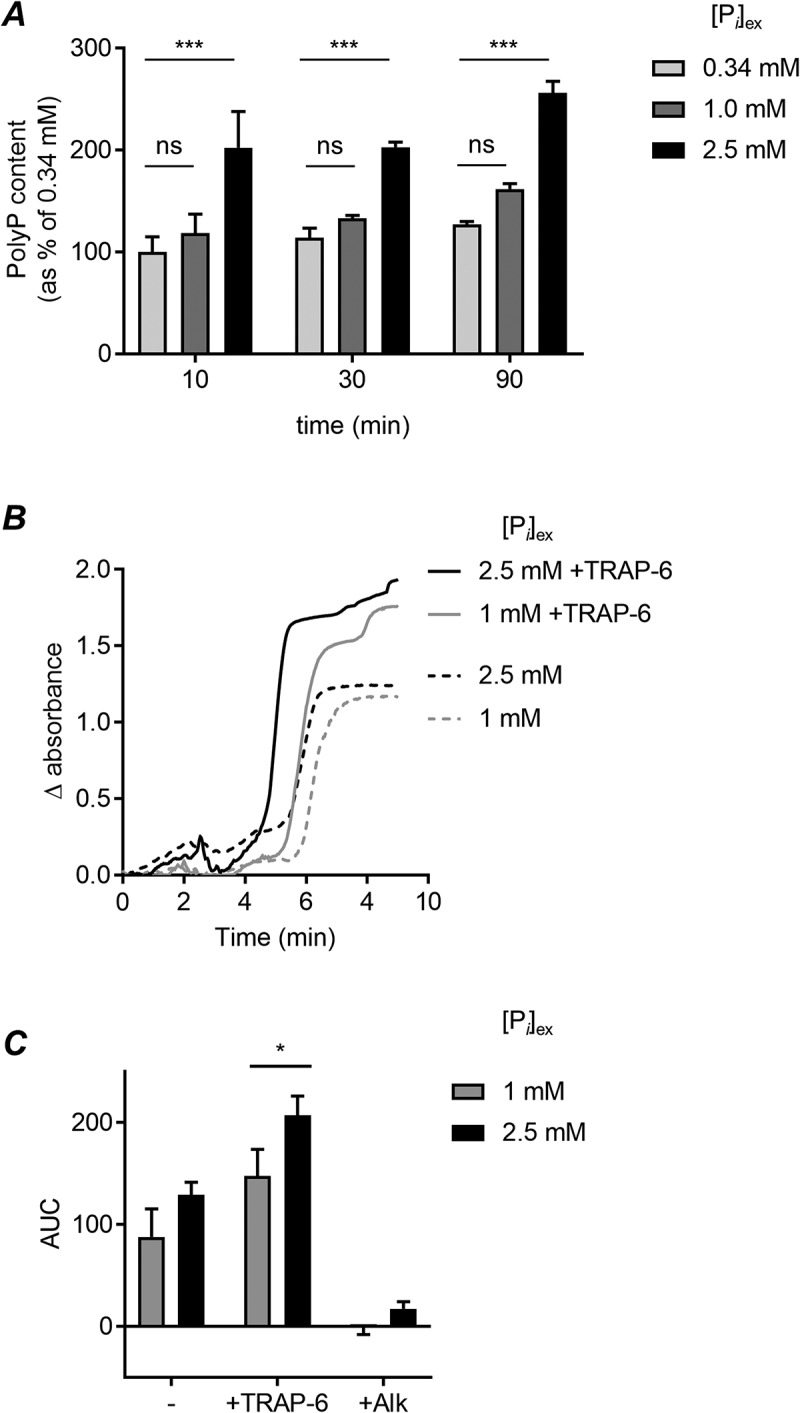
High extracellular phosphate increases platelet PolyP and clotting in PRP

Platelet PolyP content was significantly increased by incubating platelets in high [P*_i_*]_ex_ (2.5 mM) (Figure 1a). This effect was rapid, with a significant increase detected within 10 minutes. In contrast, the PolyP content of platelets was not significantly different between platelets incubated in low (0.34 mM) and physiological (1 mM) [P*_i_*]_ex_. This suggests that platelets dynamically respond to pathological elevations in [P*_i_*]_ex_ by increasing their PolyP content.

(a) Washed platelets were incubated in HEPES-buffered saline containing different concentrations of inorganic phosphate ([P*_i_*]) for the times indicated. PolyP was extracted using silica spin columns. Any DNA and RNA were degraded with DNAase and RNAase and PolyP quantified by DAPI staining. PolyP content was normalized to the PolyP content of platelets incubated with 0.34 mM P*_i_* for 10 minutes. Data are mean ± s.e.m. (n = 4). *** *p* < .001 for indicated comparison (RM 2-way ANOVA); ns, not significant. (b) Representative trace of clotting in platelet-rich plasma (PRP). CaCl_2_ was added at time = 0 to trigger coagulation of citrated PRP. Some platelets were stimulated with TRAP-6 (10 μM). An increase in absorbance (405 nm) indicated clotting. (c) Area under curve (AUC) of PRP clotting, in the absence (-) or presence of TRAP-6 stimulation. ‘Alk’ is alkaline phosphatase. Data are mean ± s.e.m (n = 4). * *p* < .05 for indicated comparison (RM 2-way ANOVA)

Clot turbidometry was used to investigate whether the increased PolyP content of platelets influences coagulation. Platelet-rich plasma (PRP) was prepared from blood drawn into sodium citrate (3.2%). NaH_2_PO_4_ was added to increase [P*_i_*]_ex_, using the malachite green phosphate assay titrate the NaH_2_PO_4_ required to elevate [P*_i_*]_ex_ to 2.5 mM. After 90 minutes, coagulation was initiated by addition of CaCl_2_ (20 mM). Clot turbidity was measured absorbance (405 nm) in a microplate reader without shaking (Figure 1b shows representative traces). The area under the curve (AUC) was measured. As expected, addition of CaCl_2_ triggered clotting of citrated PRP, whereas citrated PRP in the absence of additional CaCl_2_ did not begin to clot (data not shown). CaCl_2_-triggered clotting under these conditions (no exogenous tissue factor) was dependent on PolyP as it was abolished by the addition of alkaline phosphatase (Figure 1c). There was no significant difference in clotting at the two levels of [P*_i_*]_ex_. However, if samples where platelets were stimulated with the PAR1 agonist, SFLLRN-amide, to promote platelet activation and granule secretion, the AUC was significantly increased when [P*_i_*]_ex_ was increased to 2.5 mM (Figure 1b-c). Together, these data suggest that high [P*_i_*]_ex_ promotes the procoagulant behavior of stimulated platelets by increasing PolyP levels.

**Figure 2.**
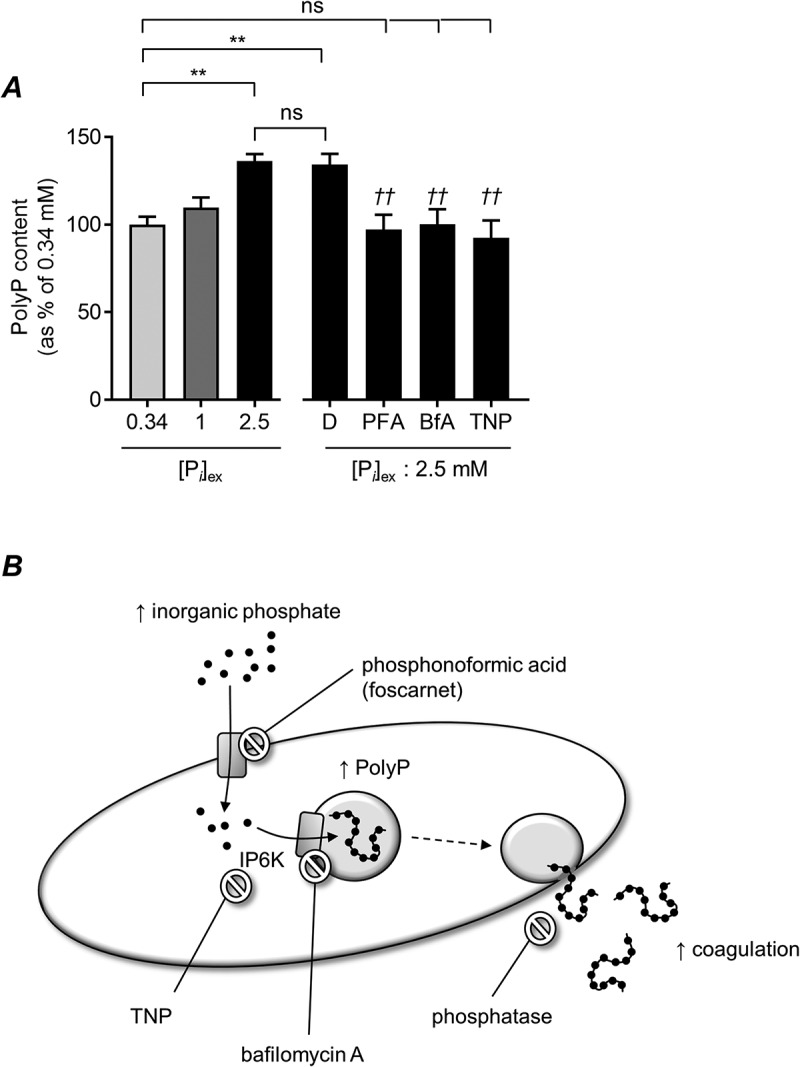
Regulation of PolyP levels by high [P*_i_*]_ex._

To investigate whether P*_i_* must enter platelets to increase PolyP levels, we used phosphonoformic acid (PFA; 1 mM) to block phosphate transporters. Pre-treatment with PFA prevented the increase in PolyP in response to high [P*_i_*]_ex_ (Figure 2a), indicating that P*_i_* must enter platelets rather than act on an extracellular site.

(a) Washed platelets were incubated in the indicated concentration of Pi for 90 minutes and PolyP levels measured as in Figure 1. Where indicated, platelets were treated with phosophonoformic acid (PFA; 1 mM), bafilomycin A (BfA; 10 μM), IP6K inhibitor (TNP; 10 μM) or the vehicle, DMSO (D), for 30 minutes. Data are mean ± s.e.m. (n = 4). ** *p* < .01 for the indicated comparison (ns, not significant); *†† p* < .01 for indicated drug treatment compared to DMSO-treated platelets. (b) Proposed mechanism for how increased extracellular inorganic phosphate (hyperphosphatemia) leads to increased platelet PolyP content, which can then be released to enhance coagulation.

V-type ATPases and inositol hexakisphosphate kinase 1 (IP6K1) have been previously implicated in PolyP synthesis and storage in platelets [4,13]. The increase in PolyP in response to high [P*_i_*]_ex_ was also inhibited by bafilomycin A, an inhibitor of V-type ATPases, and TNP, a potent and selective inhibitor of IP6K1 (Figure 2a).

## Discussion

Our study shows that platelets synthesize PolyP in response to high [P*_i_*]_ex_. We propose that Pi enters platelets through phosphate transporters and is converted to PolyP and stored in dense granules in a mechanism dependent on IP6K and V-type ATPases (Figure 2b). This mechanism may protect platelets from high [P*_i_*]_cyt_ load during periods of hyperphosphatemia. It also shows that PolyP levels in platelets are not fixed during platelet biogenesis but can rapidly fluctuate in response to changes in their environment. However, the stored PolyP is released when platelets are activated, increasing coagulation.

The increase in PolyP content was inhibited by PFA, which inhibits phosphate transporters. PFA also inhibits viral DNA polymerase, underpinning its clinical use as an antiviral (where it is called foscarnet). Although the concentration used in our study is high, it is possible that similar concentrations can be achieved in patients[14], although blocking phosphate transporters (including in the kidney) is unlikely to be useful in hyperphosphatemia. Instead, blocking the PolyP synthesis pathway may be of more benefit.

How PolyP is synthesized and regulated in mammalian cells is not well understood. In fungi and some protists, such as *Trypanosoma* and *Leishmania*, PolyP synthesis requires a vacuolar transporter chaperone complex. PolyP that is synthesized at the cytoplasmic face of acidocalcisome-like organelles by this complex is translocated into the organelle lumen. A transmembrane proton gradient generated by V-type ATPase activity is also required[15]. Notably, platelet polyphosphate is stored in dense granules, a secretory lysosome-like organelle that has an acidic lumen and is similar to acidocalcisomes[4]. Since bafilomycin prevented the increase in PolyP, platelets may use the proton gradient across the dense granule membrane to synthesize and accumulate PolyP.

IP6K1 also regulates PolyP levels in eukaryotic cells. Although normal function of IP6K1 is to synthesis 5-diphosphoinositol pentakisphosphate (IP_7_) from inositol hexakisphosphate (IP_6_), Ip6k1-deficient yeast has substantially reduced levels of PolyP, suggesting a link between the metabolism of PolyP and inositol pyrophosphates. Similarly, the PolyP level in platelets from *Ip6k1^−/-^* mice is half that of wild-type platelets[13]. Plasma clotting time was prolonged in the *Ip6k1^−/-^* mice. Tail bleeding time was also increased, and the mice were protected in a model of pulmonary embolism. Consistent with this, the increase in PolyP in response to high [P*_i_*]_ex_ was inhibited by TNP.

It would be interesting to investigate whether platelets from patients with hyperphosphatemia may have increased PolyP levels and whether this contributes to abnormal coagulation. Since it is possible to block the increase in PolyP levels, as suggested by our data, or the pro-thrombotic effects of PolyP[2], PolyP is a potential pharmacological target to reduce the pathophysiology associated with hyperphosphatemia.
